# Opportunities and challenges in automated coding of electronic health records: a pilot study for rare disease registries

**DOI:** 10.3389/fdgth.2026.1807607

**Published:** 2026-07-08

**Authors:** Damiano Paoli, Marcella Lanza, Federico Banchelli, Manila Boarini, Stefano Borghi, Luca Sangiorgi, Marina Mordenti

**Affiliations:** 1 Department of Rare Skeletal Disorders, IRCCS Istituto Ortopedico Rizzoli, Bologna, Italy; 2 Department of Innovation in Healthcare and Social Services, Emilia-Romagna Region, Bologna, Italy; 3 Dilaxia S.p.A., Castel Maggiore (BO), Italy

**Keywords:** artificial intelligence, automated coding system, disease registry, ICD, precision medicine, rare diseases

## Abstract

Accurate medical coding is essential for disease registries, particularly in the context of rare conditions. Manually transforming electronic health records data into standardized codes is time-consuming and resource intensive. This study evaluates an automated coding system using synthetic health records data and explores the potential benefits and challenges of introducing this tool into rare diseases registries activities. We developed a hybrid architecture combining a symbolic component with medical knowledge graphs and an ensemble of three widely used Large Language Models with a critical review mechanism. Ninety-nine synthetic Italian-language clinical reports were coded by the system. Subsequently, a multidisciplinary expert panel performed a double-coding validation of extracted terms, categorizing automated results into four groups: correct, incorrect, inaccurate, or missing codes. The system extracted a total of 479 terms (264 diagnosis codes and 215 procedure codes) mapped to ICD-9-CM classification. The expert panel, considered as the gold standard, identified 500 terms (302 diagnosis codes and 198 procedure codes). Chi-square analysis highlighted statistically significant differences between diagnosis and procedure coding in at least one of the four groups of results (*p*=0.001). The system achieved an accuracy of 70.53% for diagnoses, compared to 78.28% for procedures. Additionally, the relative frequency of the various incorrect codes is generally consistent and uniform, except for two incorrect procedure codes that are particularly prevalent. Considering all the findings, we critically point out the potential contribution and impact of an automated coding system into rare diseases registries process, examining the benefits and barriers that could facilitate or hamper progress in this specialized field. The automated coding system demonstrated reasonable accuracy with health records synthetic data. Key challenges include limited ICD-9-CM codes, particularly for rare diseases, overreliance on nonspecific residual codes, and tendency to generate details not present in reports. Opportunities for future improvement may include adopting the ICD-10/ICD-11 classification, implementing reliability metrics and a multi-ontology approach, thus promoting data interoperability according to FAIR principles. An automated coding system, properly improved, may have an essential impact for rare disease registries and are welcomed by several initiatives such as the EHDS.

## 1 Introduction

Accurate coding of medical diagnoses and procedures is critical in clinical practice and in cross-border research endeavors (1–3). Medical doctors and researchers face this aspect, on a daily basis, both at the hospital level and internationally. Currently, Italy and many other countries use the International Classification of Diseases (ICD), the worldwide recognized coding system for healthcare, developed by WHO (4). The ICD is a hierarchical tree structure classification system that plays a key role in general health management and in several aspects of downstream applications, including medical discharge forms, data retrieval, research studies, disease registries and other epidemiological evaluations (5–7). Over time, the ICD classification system has undergone several revisions aimed at improving coding granularity and clinical representation. ICD-9-CM, widely adopted in clinical and administrative settings for many years, was followed by ICD-10, which introduced greater specificity and a more detailed coding structure, and more recently by ICD-11, designed to support digital health applications, interoperability, and more advanced semantic representations. Nevertheless, in addition to ICD, there is a large number of available vocabularies, ontologies, with more emerging daily.

Disease registries (DRs), as sources of structured data, are of significant value and support natural history studies, foster clinical and epidemiological research, facilitate therapeutic interventions and promote regulatory assessments (8–10). DRs play a pivotal role in the pursuit of evidence-based medicine and personalization of care and can greatly benefit from appropriate and accurate coding (11). The utilization of codification standards is strongly endorsed to support data capturing in a broad range of clinical conditions (12–14). In the context of rare diseases (RDs), the importance of proper coding is further accentuated. RDs are a group of conditions primarily defined by their low prevalence and high phenotypic and genotypic diversity (15, 16) with information often scattered across various hospitals and institutions. These conditions can benefit even more than more common ones of a proper coding system, capable of managing the multifaceted aspects of RDs nature. In fact, the European Commission has promoted numerous initiatives for rare diseases, supported projects and proposed guidelines to encourage and support standardized codification (17–20). Nevertheless, the process of transforming electronic health record (EHR) contents into data captured in disease registries nowadays relies on expert personnel with a strong background on diseases. These personnel meticulously navigate several sources to identify the appropriate code for each distinct diagnosis, sign, symptom, procedure, or therapy.

Artificial Intelligence (AI) is an evolving branch of computer science that focuses on creating intelligent agents capable of performing tasks that typically require human intelligence, such as learning, reasoning, problem-solving, and language understanding (21). The automated prediction of codes nowadays represents a rapidly evolving topic in the field of artificial intelligence, albeit one that remains challenging. Several researchers faced this approach for both common and rare diseases, examining AI potentialities in several classifications and ontologies, even if the ICD still represents one of the most studied resources (22–24). However, the majority of these research studies are designed to primarily support healthcare services, with the objective of introducing AI coding into clinical practice. AI models for automated coding are undoubtedly a step forward also in DRs context, promoting natural history studies, disease evolution studies as well as planning and following clinical trials.

The objective of the present study is to describe the opportunities and challenges associated with introducing automated coding into the routine activities of rare disease registries, based on findings derived from a pilot study conducted on synthetic EHR data. The proposed approach was evaluated considering the perspective of professionals working in the field of rare disease registries and its potential applicability within this setting.

## 2 Materials

### 2.1 Coding assistant architecture

The automated coding system (ACS) proposed in this study adopts a parallel hybrid architecture that combines two complementary artificial intelligence paradigms: the symbolic approach based on explicit knowledge and the subsymbolic approach founded on Large Language Models (LLMs) (25, 26).

The algorithm processes each clinical document through two independent coding components operating in parallel:

1. Symbolic Component (Expert.ai) (27): a symbolic artificial intelligence system that utilizes medical knowledge graphs and explicit inference rules
2. Subsymbolic Component: an ensemble of three LLMs with critical review mechanism

#### 2.1.1 Symbolic coding component

The Expert.ai-based component leverages a symbolic AI architecture, designed to provide transparent and interpretable coding decisions. This component operates through the following mechanisms:

**Knowledge graph structure:** the system employs a comprehensive medical knowledge graph that encodes relationships between clinical concepts, ICD-9-CM codes, and their hierarchical organization.
**Rule-based inference engine:** the coding process relies on explicit “if-then” rules that formalize clinical coding guidelines.

A fundamental advantage of the symbolic approach is its inherent interpretability. Each coding decision can be traced back to specific rules and knowledge graph traversals.

Mitigation of Hallucinations: unlike neural approaches, symbolic systems do not generate outputs probabilistically. The deterministic nature of rule-based inference significantly reduces the risk of “hallucinations”, instances where the system produces plausible but factually incorrect codes not supported by the input text.

#### 2.1.2 LLM-based coding component

The Anthropic Claude Sonnet (28) component represents the subsymbolic paradigm, leveraging deep learning architectures trained on extensive corpora of medical literature and clinical documentation.

It is important to emphasize that the corpora used to train the subsymbolic component based on LLM are not publicly known. The companies that produce these models do not disclose detailed information about the datasets used during the training phase. As a result, it is not possible to reliably verify the coverage, quality, or representativeness of the clinical data on which the model is based.

This component offers distinct capabilities:

**Contextual understanding:** LLMs excel at capturing nuanced linguistic patterns and contextual relationships that may be difficult to encode in explicit rules (29).
**Semantic flexibility:** the neural architecture enables the system to generalize beyond explicitly programmed patterns

The subsymbolic component employs an ensemble architecture consisting of four LLMs:

**Independent coding phase:** three widely used language models operate independently on the same clinical text:

1. Google Gemini (30)
2. OpenAI GPT-4 (31)
3. Anthropic Claude Sonnet (28)

Each model generates ICD-9-CM code predictions based on its interpretation of the clinical narrative. This multi-model approach leverages the distinct architectures, training data, and reasoning patterns of each system.

**Critical Review Phase:** a fourth model (Google Gemini) operates as a critical reviewer, systematically analyzing the outputs generated by the three coding models. This component evaluates the clinical correctness and relevance of each proposed ICD-9-CM code, assessing clinical validity to verify that assigned codes are appropriate given the provided clinical documentation, logical coherence to identify incompatibilities between proposed codes (such as mutually exclusive diagnoses), and textual support to confirm that each code is actually justified by elements present in the clinical text. Codes deemed incorrect are eliminated from the final output, producing a refined set of codes representing the validated consensus of the models. This critical review process significantly reduces the risk of hallucinations and coding errors that could arise from direct application of unfiltered outputs from individual LLMs.

#### 2.1.3 Code integration strategy

The final code assignment combines outputs from both the symbolic and subsymbolic components through a union operation rather than intersection. This design choice addresses a fundamental limitation: the Expert.ai symbolic system does not contain rules covering the entire ICD-9-CM classification system.

The union approach ensures:

- Maximum coverage: codes identified by either component are included in the final output
- Complementary detection: the symbolic system captures well-defined patterns while LLMs identify cases beyond the rule base
- Robustness: system limitations in one component do not prevent code assignment by the alternative pathway

This integration strategy prioritizes sensitivity (comprehensive code detection) while accepting the risk of reduced specificity, with the critical review mechanism providing quality control within the LLM ensemble.

### 2.2 Synthetic data and AI results

As defined by Jordon et al., synthetic data are “data that has been generated using a purpose-built mathematical model or algorithm, with the aim of solving a (set of) data science task(s)” (32). In the present study, a set of 99 machine-generated synthetic data, mimicking EHR clinical reports in Italian natural language, was generated. This generation of synthetic reports was driven by the necessity to encompass a broad spectrum of clinical conditions and surgical, diagnostic, and therapeutic procedures with the objective of providing representative examples of real-world data. This approach was undertaken to circumvent potential ethical and legal concerns that might arise from the collection and utilization of real reports according to European and National Regulations (33). Each synthetic report contained text referring to a set of diseases and medical procedures.

For each clinical term of interest, the ACS generated a “code” and a “description”, following the structure of the ICD-9-CM, and kept trace of the “text reference” (in Italian), that represents the specific text related to the code, even if the AI model evaluated the entire report text for individuating the appropriate code. Both the automated coding system and data expert panel used the ICD-9-CM (2007 version – Italian translation, the one officially in use for clinical data coding), that included all the following codifications: diagnosis codes comprising V codes (factors influencing health status or encounters for reasons other than illness or injury, such as vaccinations, screenings, or aftercare) and E codes (external causes of injuries or poisoning, including the circumstances, intent, and location of the event), and procedure codes. ICD-9-CM, despite the availability of newer revisions, remains routinely adopted within the Italian healthcare context, reflecting established expertise and daily coding practices. Although more recent classification revisions are available, covering a broader range of terms and codes, ICD-9-CM may still retain practical value for historical and retrospective data sources, particularly in contexts such as chronic and hereditary diseases, disease registries, and long-term clinical datasets, where historical medical records frequently need to be retrieved and integrated.

Following an ACS output example:

*{'diagnoses': [{'code': '340', 'description': 'Multiple sclerosis', 'text reference': 'Paziente affetta da sclerosi multipla.'}*

Of the entire resulting content, the diagnosis or procedure code was the only parameter to be evaluated, as description and text reference were only provided to facilitate data expert evaluations.

### 2.3 Methods

#### 2.3.1 The data expert panel

A multidisciplinary panel of data experts from the IRCCS Istituto Ortopedico Rizzoli (IOR), a highly specialized hospital and research institute in the field of orthopedics and traumatology, was established. The panel is comprised of four data experts with different backgrounds and areas of expertise, including a clinical researcher who acts as the registry manager (MM), two data managers who participate in all the data activities (MB and ML), from data inputting to conducting research on it, and a data curator who is primarily involved in data management (DP). All of the data experts are highly skilled in coding clinical features and working on codification procedures. Indeed, to populate DRs, these experts navigate several sources on a daily basis to select the appropriate code for each distinct value captured i n RDs registries. Furthermore, two additional experts were involved in this study, to provide support to the data expert panel: a physician (LS) with a multidisciplinary background intended to provide support for the clinical evaluation of specific, complex diagnoses and procedures and a biostatistician (FB) with a strong background in epidemiology, and clinical data coding with ICD-9-CM.

#### 2.3.2 The pilot study

The pilot study was composed of a preliminary assessment performed on 10 synthetic reports that then were incorporated into a set of reports consisting of 99 cases. All reports were fully synthetic and generated from occupational medicine reports to emulate realistic clinical scenarios. Occupational medicine was selected as the source domain because it provides a broad spectrum of diagnoses and procedures, making it highly representative for evaluating coding performance across heterogeneous clinical scenarios. This choice was also motivated by the limited suitability of ICD-9-CM for rare diseases, the primary field of interest of our research, where coding granularity and representativeness are often inadequate.

During both the preliminary feasibility assessment and the pilot study results, the expert panel has performed non-blinded double coding, starting from the diagnosis and procedure codes generated by the automated coding system.

The preliminary assessment was intended to evaluate key aspects related to study feasibility s. These included the evaluation of the synthetic reports and their representativeness of real reports, the availability of project requirements in terms of resources and expertise, and the estimation of the time required for the overall process. The absence of real-world data avoided ethical and legal constraints, thereby facilitating the feasibility assessment. The ten synthetic reports initially analyzed were subsequently incorporated into the pilot study, resulting in a final dataset of 99 reports.

**Figure 1:**
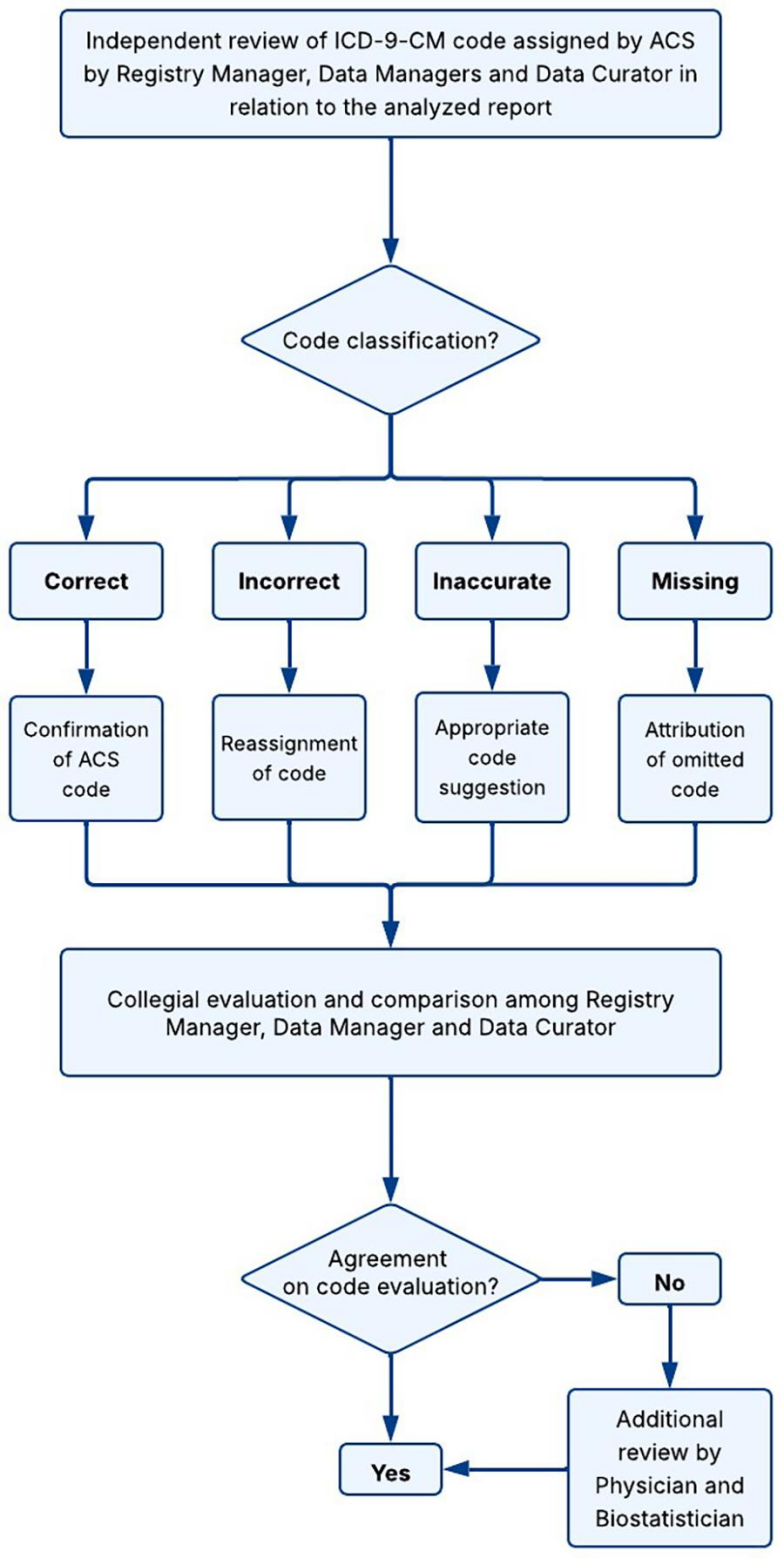
Workflow of the evaluation process adopted in the pilot study. AI-generated ICD-9-CM codes were independently reviewed by data experts and subsequently discussed through a collegial assessment process to resolve discrepancies and complex cases. When required, additional clinical and methodological support was provided by a physician and a biostatistician. The final evaluation resulted in code classification and confirmation or reassignment of the ACS output.

The pilot study followed a multi-step evaluation process. Firstly, the synthetic reports were distributed among the data experts, allowing each evaluator to independently assess a subset of reports. During the evaluation process, AI-generated codes were assessed using official ICD-9-CM digital platforms and supporting documentation released by the Italian Ministry of Health (Ministero del Lavoro, della Salute e delle Politiche Sociali), as official tools for coding assessment and decision-making. Each evaluation was subsequently reviewed by a second data expert to ensure consistency and reduce potential discrepancies. Individual evaluations were then followed by a collegial review process aimed at resolving potential inconsistencies in coding decisions among data experts. Whenever additional clinical or methodological support was required, the physician and biostatistician provided expert guidance, offering support in the interpretation of complex clinical cases and methodological issues encountered during the coding evaluation process. Finally, the coding outcome was classified according to predefined categories, resulting either in confirmation of the ACS code assignment or reassignment of a more appropriate code. The overall evaluation and decision-making process is summarized in the flowchart shown in Figure 1.

#### 2.3.3 Type of results

The automated coding results were then validated by the expert panel and those that were determined to be correct were designated as such. As an example of correct code:

*{'diagnoses': [{'code': '250.00', 'description': 'Diabetes mellitus without mention of complication, type II or unspecified type, not stated as uncontrolled', 'text reference': 'Uomo con diabete mellito tipo 2, in buon compenso glicemico.' (Male patient with type 2 diabetes mellitus, with good glycemic control)}]*

Errors were then organized into three distinct groups, each with a different level of severity.

These groups were as follows:

(a) incorrect result: the AI code is erroneous, with the consequence being a direct effect on clinical impact;
(b) inaccurate result: the AI coding is imprecise, with the consequence being a minor effect on clinical impact;
(c) missing code: the automated coding system has not considered a clinical term.

Some examples are presented in Table 1.

**Table 1:** Examples of errors. The table presents two examples for each error type. The first line provides text references, the second line the system coding output and the final line the expert panel output.

Entry	Incorrect result	Inaccurate result	Missing code
Text reference 1	text reference: ‘discopatia L5-S1’ (discopathy L5-S1)	text reference: ‘incidente stradale’ (motor vehicle accident)	text reference: ‘Paziente con lombalgia cronica’ (patient with chronic lumbago)
Automated Coding output 1	code: ‘722.73’, description: ‘Intervertebral disc disorder with myelopathy, lumbar region’	code: ‘E819.0’, description: ‘Motor vehicle traffic accident of unspecified nature injuring driver of motor vehicle’	code: ‘724.2’, description: ‘Lumbago’
Panel output 1	code:*'*722.52*'*, description: ‘Degeneration of lumbar or lumbosacral intervertebral disc’	code:‘E819.9’, description: ‘Motor vehicle traffic accident of unspecified nature injuring unspecified person’	code:‘724.2’, description: ‘Lumbago’ code:‘338.29’ description: ‘Other chronic pain’
Text reference 2	text reference: ‘Gastrite atrofica autoimmune dal 2023’ (Autoimmune atrophic gastritis since 2023)	text reference: ‘A febbraio 2023 diagnosi di Bronchite cronica’ (Chronic bronchitis diagnosed in February 2023)	text reference: ‘Visita fisiatrica con programmazione di ciclo riabilitativo’ (Physiatric consultation with prescription of a rehabilitation program)
Automated Coding output 2	code: ‘535.4’, description: ‘Other specified gastritis’	code: ‘491.20’, description: ‘Obstructive chronic bronchitis without exacerbation’	code: ‘93.89’, description: ‘Rehabilitation procedure, not elsewhere classified’
Panel output 2	code: ‘535.1’ description: ‘Atrophic gastritis’	code: ‘491’ description: ‘Chronic bronchitis’	code: ‘93.89’, description: ‘Rehabilitation procedure, not elsewhere classified’ code: ‘89.7’ description: ‘General physical examination’

#### 2.3.4 Statistical analysis

A descriptive analysis of the coding results was conducted by calculating and comparing the percentages of the codes in each group out of the total number of codes identified by the experts' panel.

The Chi-squared (*χ*²) test was used to evaluate differences in the distribution of categorical data across groups. Results were considered statistically significant with a two-tailed *p*-value < 0.05.

The Gini concentration index (*R_G_*) was calculated using the following formula:

$$R_{G}=1-2\frac{\sum_{i=1}^{n-1}\;Q_{i}}{n-1}$$

where *Q_i_* represents the cumulative relative frequencies and *n* is the number of units considered. This indicator provides a measure of the concentration of a variable, i.e., how unequally a variable is distributed among the units considered. A value of 0 indicates maximum equity (the variable is distributed equally among all units), while an increase towards 1 suggests an increase in the concentration of the variable, and therefore a situation of inequality in distribution.

All statistical analyses were performed using StataNow/SE version 18.5.

## 3 Results

Four hundred and seventy-nine terms were extracted from the synthetic reports and mapped on ICD-9-CM by the ACS, comprising 264 diagnosis codes and 215 procedure codes. Subsequently, the panel of data experts was able to identify 500 terms, including 302 diagnosis codes and 198 procedure codes. Each synthetic report comprised a set of diagnosis and procedure codes, whose number ranged from 2 to 9.

**Figure 2:**
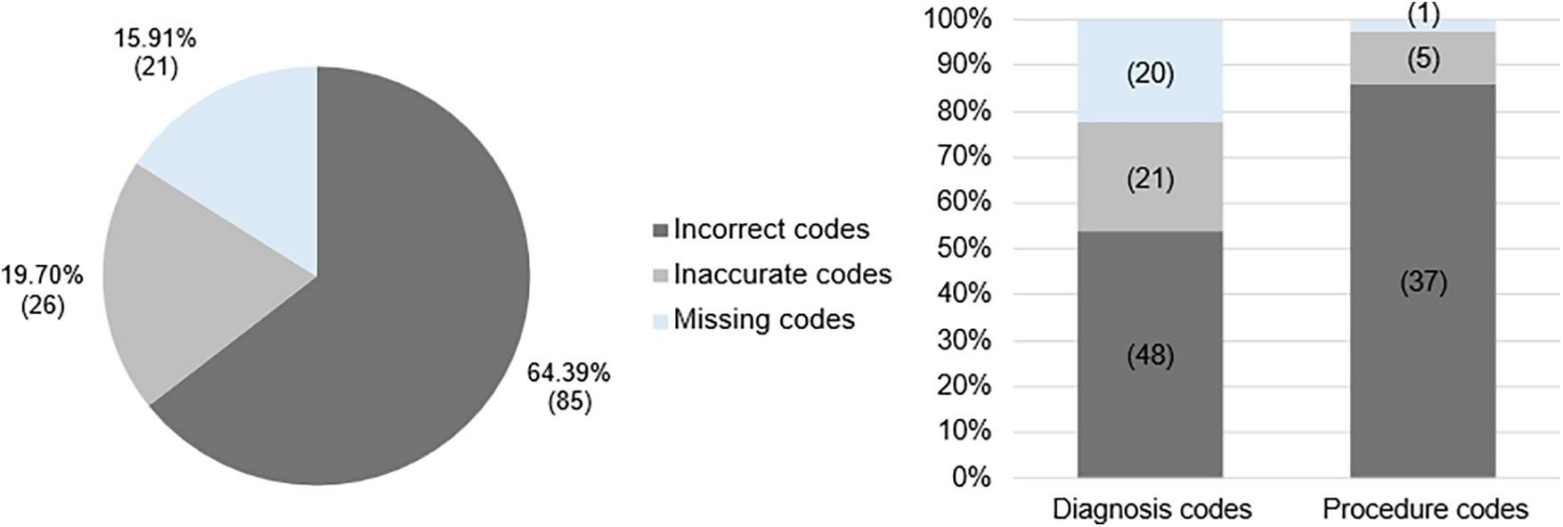
Distribution of the detected erroneous codes. (Left) Overall, 85 incorrect codes (64.39%) constitute the majority, compared to 26 inaccurate (19.70%) and 21 missing codes (15.91%). (Right) More specifically, among the diagnosis codes, 48 incorrect codes (53.93%), 21 inaccurate codes (23.60%), and 20 missing codes (22.47%) were found, whereas 37 incorrect codes (86.05%), 5 inaccurate codes (11.63%), and 1 missing code (2.33%) were observed among the procedure codes.

Overall, evaluating the output of the automated coding system, correct codes are 368 (73.60%), incorrect codes are 85 (17.00%), inaccurate codes are 26 (5.20%), while the remaining 21 terms are missing codes (4.20%). Figure 2 shows the overall proportion of error codes and divided by diagnosis and procedure code types.

Since data expert panel output has been considered the gold standard, 500 represented the actual number of codes present in the totality of synthetic reports, thus also including missing codes. Looking more deeply into the results obtained by the automated coding system and reviewed by humans, correct codes include 213 diagnosis codes (70.53%) and 155 procedure codes (78.28%); incorrect codes consist of 48 diagnosis codes (15.89%) and 37 procedure codes (18.69%), while inaccurate codes comprise 21 diagnosis codes (6.95%) and 5 procedure codes (2.52%); finally, the missing codes are divided into 20 diagnosis codes (6.62%) and 1 procedure codes (0.5%).

The investigation of significance was conducted through the utilization of a chi-square test, which was employed to analyze the discrepancy between procedure correct codes and diagnosis correct ones (Table 2). The study revealed no statistically significant difference (*p* = 0.054) but suggested that the percentage of correct codes among procedure codes tends to be higher than that among diagnosis codes. However, this observation requires further investigation using a more extensive dataset.

**Table 2:** Comparison of the proportion of correct codes between diagnosis and procedure codes. The table shows absolute frequencies of erroneous and correct codes, with the row percentages in brackets. The erroneous codes include incorrect, inaccurate, and missing codes. The correct codes are 213 (70.53%) for diagnosis codes and 155 (78.28%) for procedure codes. The chi-square test did not show a statistically significant difference between diagnosis and procedure codes (*p* = 0.054).

Code's type	Erroneous codes	Correct codes	Total
Diagnosis Codes	89 (29.47%)	213 (70.53%)	302
Procedure Codes	43 (21.72%)	155 (78.28%)	198
Total	132 (26.40%)	368 (73.60%)	500

To investigate this aspect, we broadened the spectrum of analysis. A single chi-square test was used to confront diagnosis codes and procedure codes, considering each of the four groups of codes. The results showed a statistically significant difference (*p* = 0.001), suggesting that the proportion of procedure and diagnosis codes actually differs in at least one of the four groups considered.

**Figure 3:**
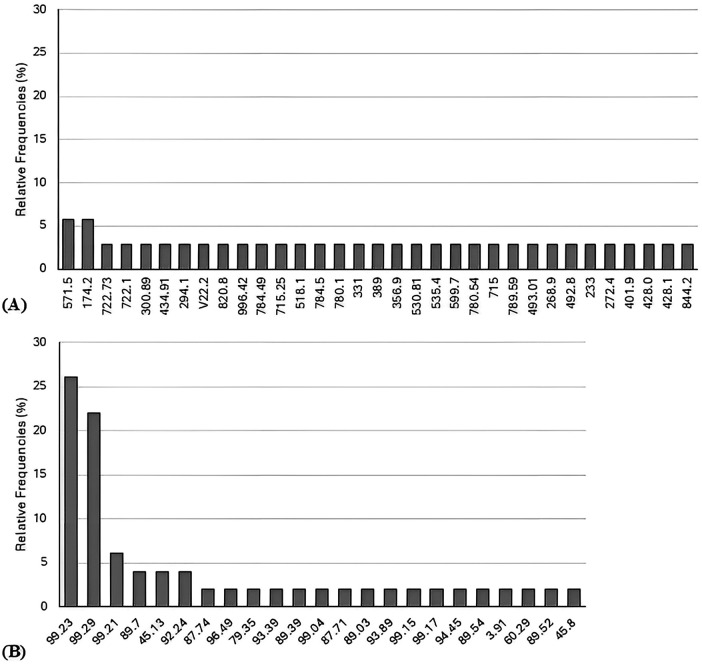
Relative frequency of incorrect codes given by the automated coding system. **(A)** The relative frequencies of the incorrect diagnosis codes are basically homogeneous. Almost all appear only once (2.86%), except for “571.5” and “174.2”, which were incorrectly assigned twice (5.71%) by the automated coding system; **(B)** looking at the relative frequencies of incorrect procedure codes, there is an evident gap between two groups of codes. On the one hand, some codes have low frequency, such as “87.74” assigned once (2%) or “99.21” assigned three times (6%); on the other hand, two codes were selected with a high frequency. In fact, code “99.29” occurred 11 times (22%) and “99.23” occurred 13 times (26%). These last two codes were assigned by the automated coding system with a frequency that is 4 to 13 times higher.

The relative frequency of the various incorrect codes given by the system (Figure 3) is generally constant and homogeneous, except for two recurrent codes: a) “99.23: injection of steroid” and b) “99.29: Injection or infusion of other therapeutic or prophylactic substance”. These codes belong to the “other non-operative procedures” section, containing the most nonspecific and residual procedure codes provided by ICD-9-CM. As shown in the bar graph (Figure 3B), “99.23” and “99.29” have a relative frequency of 26% and 22% respectively, which means they are up to 13 times more frequent than other incorrections among procedure codes assigned.

To give a measure of the inequality in the distribution of incorrect diagnosis codes and incorrect procedure codes, we used the Gini concentration index (R_G_). The distribution of incorrect diagnosis codes is characterized by an R_G_ = 0.11, indicating a highly equal distribution. Conversely, the distribution of incorrect procedure codes gave an R_G_ = 0.73, showing how the redundancy of codes “99.23” and “99.29” makes it unequal. A similar analysis could be informative and completive if referred to missing codes. Since there is only one missing procedure code, the calculation was solely performed for the 20 missing diagnosis codes, resulting in an R_G_ = 0.03. Hence, these codes also have a highly equal distribution, with no evident disproportion.

## 4 Discussion

### 4.1 Input data and results

The automated coding system worked on clinical reports obtained from synthetic data. The use of synthetic data is becoming increasingly widespread to bypass the ethical, legal, and logistical limitations associated with real patient data. In fact, this type of data is artificially generated to reproduce the statistical and semantic characteristics of real data, without containing any directly identifiable information (34). This fundamental feature makes it possible to reduce the risk of violating the General Data Protection Regulation (GDPR) (35), in force in the European Union, or patient privacy, since synthetic data does not derive directly from real individuals and presents minimal or no risk of re-identification (36). Therefore, synthetic data undoubtedly simplifies research activities, reducing the need for pseudonymization procedures or ethical approvals (37).

Another practical and tangible advantage is the acceleration of the data collection process. When using synthetic data, there is no need to wait for the collection and authorization of large real clinical datasets. In this context, this enables the quick training, testing, and optimization of algorithms in the pre-training phase, prior to moving on to real data.

As shown in the results, the synthetic reports contained more diagnoses than procedures. The expert panel mapped 302 diagnosis codes and 198 procedure codes, while the ACS identified 264 diagnosis codes and 215 procedure codes. The numerical difference between these two coding results is given by the 21 missing codes, omitted by the system, while mapped by humans. Considering the system efficiency related to diagnoses and procedures coding, the chi-square test showed only a trend (*p* = 0.054) of the percentage of correct procedure codes (78.28%) being higher than that of correct diagnosis codes (70.53%). This means that the ACS is able to correctly code diagnoses and procedures with a good degree of accuracy in both cases and, at least in this aspect, does not present any critical issues in one category of code over the other. This trend should be investigated in a wider set of reports for confirmation. Therefore, the ACS demonstrated moderate accuracy with synthetic health records. The accuracy can be considered acceptable for high-scale clinical data coding in registries, but will often require double-check by a data expert. This performance may be considered acceptable in light of the complexity of automated coding tasks and the use of ICD-9-CM, which offers lower granularity and specificity than ICD-10 and may therefore pose additional challenges for coding diagnoses and procedures from free-text clinical reports. Higher performance values have been reported in the literature for automated coding systems, with some ICD-10-based studies reporting accuracy levels close to or above 90% (38). However, these findings were often obtained using larger datasets, more granular coding systems, constrained coding scenarios, and tasks limited to primary diagnosis coding. A review of the field has also highlighted the substantial heterogeneity in datasets, coding strategies, and evaluation approaches adopted across studies (39). Such methodological differences should be considered when comparing performance with ICD-9-CM-based approaches and pilot evaluations conducted in more heterogeneous settings. Importantly, the present study was not primarily designed as a performance-driven evaluation focused on maximizing coding accuracy. Instead, its main objective was to explore the potential role of automated coding systems as supportive tools for researchers and professionals involved in rare disease registries.

Furthermore, it has been seen how at least one of the four groups (correct, incorrect, inaccurate, and missing codes) has statistically different proportions between diagnosis codes and procedure codes (*p* = 0.001). This suggests that, setting aside the correct codes already discussed, the ACS commits at least one type of error more often when coding diagnoses or procedures. Given the potential significance of ICD-9-CM codes as the primary source of information for clinicians and researchers, the presence of erroneous codes can have a substantial impact on clinical evaluation, treatment plans and research studies.

The incorrect codes that garner the most attention constitute 86.05% of procedure erroneous codes and 53.93% of diagnosis erroneous codes. As shown in Figure 3B, the difference is largely due to two highly repeated incorrect procedure codes, “99.23: injection of steroid” and “99.29: Injection or infusion of other therapeutic or prophylactic substance”. These two codes are assigned by the ACS with a frequency that is 4–13 times higher than other procedure or diagnosis codes, a prevalence such as to give a Gini concentration index of 0.73. Contrary to what has been seen for incorrect and missing diagnosis codes, the result given by this index confirms the measure of how unequal the distribution of incorrect codes is among the procedure codes, precisely because of the two redundant codes mentioned above. Interestingly, “99.23” e “99.29” belong to the “other non-operative procedures” section, containing the most nonspecific and residual procedure codes provided by ICD-9-CM. In particular, from a clinical point of view, codes of type “99.xx” are used only when the procedure performed cannot be described by any other more specific procedural or diagnostic code, for example when it involves medical, pharmacological, or supportive procedures that are not surgical. This means that on the one hand, the ACS does not show significant differences in the correct coding of diagnoses and procedures, but on the other hand, it clearly misuses some of the procedure codes intended for coding terms that do not fit into the previous categories of ICD-9-CM.

An additional consideration concerns the selection of the resources composing the proposed architecture. The selected tools were intended as complementary. The symbolic resource was chosen for its transparency, explicit rule representation, and reduced susceptibility to hallucinations, whereas LLM-based resources were selected for their contextual understanding and semantic flexibility. Multiple LLMs were intentionally included to leverage differences in architectures, training corpora, and reasoning patterns. The goal was not to identify a single optimal resource, but rather to evaluate the feasibility of integrating complementary approaches. Future implementations may adopt alternative resources according to criteria such as interoperability, explainability, regulatory constraints, accessibility, and domain-specific performance.

Challenges and Opportunities of introducing an automated coding system in rare disease registries.

Considering the experience gathered during the pilot study, several opportunities and challenges related to the implementation of an ACS in the context of rare disease registries were identified, and an attempt was made to sift through each potential aspect.

#### 4.1.1 Expected challenges

The evaluated ACS uses ICD-9-CM, that is the Clinical Modification of the ninth revision of the ICD classification, as the reference ontology. It was released in 1979 and revised until 2015, when it was officially replaced by the ICD-10-CM for reporting. ICD-9-CM is the version adopted by the Italian National Health System and contains more than 12,400 diagnosis codes and about 3,700 procedure codes. Its structure is strictly hierarchical but rather rigid: in fact, there are no explicit semantic relationships between concepts, except through simple taxonomic groupings (40, 41). However, retroactive attempts have been made to map ICD-9-CM to semantic ontologies, for example through exposure on platforms such as BioPortal, where some code sets have been restructured to typical formats of its successors ICD-10 (OWL, Web Ontology Language) and ICD-11 (RDF, Resource Description Framework) (42, 43). Furthermore, ICD-9-CM remains the coding standard currently adopted within the Italian healthcare context, even though transition to ICD-10 is expected in the next few years. Therefore, its use was consistent with current coding practices and existing expertise within the national healthcare setting process.

Although the ICD-9-CM coding includes terms related to various diagnoses/procedures, one of the main challenges involves the scarcity of codes referring to RDs. This is compounded by the fact that in this field, given the small number of patients, the data collection process during training is severely limited (16), reducing the so-called parametric knowledge, the knowledge derived from large corpora stored in model parameters. This can become an obstacle not only in the care setting, but also in the activities of disease registries, which require standardization and structuring of data with precise terms. Another challenge is methodological nature and concerns the inclusion of a control group. This would improve the quality of evidence of studies involving clinical coding with ACS, as the presence of a control group, such as a second, blinded coding experts panel, can favor a more accurate assessment of the true coding performance.

In our validations, we found that 12.61% of the errors - intended as the sum of inaccurate and incorrect results - are terms that do not have a specific code in ICD-9-CM, nevertheless the ACS gives them a classification code. This is due to the partial absence of structured semantic relationships in ICD-9-CM above mentioned, coupled with the relative lack of terms compared to more up-to-date coding systems, aspect which limits the model's ability to anchor itself to a solid ontological “ground truth” (44). This can promote errors in automatic coding, particularly when the model connects terms with existing codes even in the absence of formal correspondences within the ICD-9-CM taxonomy.

Furthermore, we observed that in the presence of a diagnosis or procedure that does not have a specific code in ICD-9-CM, the ACS tends to select recurrent codes, as mentioned in the results section (Figure 3). This behavior was explained by Soroush et al. (45) by indicating how the statistical frequency of the code within the clinical data constituting the parametric knowledge is the model's preferred factor for attribution to the diagnosis/procedure. This means that the ACS tends to return the most “common” or frequent codes by also associating them with the above diagnoses or procedures, based on what it has learned statistically during training.

We have noticed how the ACS tends to include details about diagnoses or procedures that are not mentioned in the report but rather are an insight into the case without any real evidence. This kind of output represents 10.81% of the errors and concerns parametric knowledge. Although it helps improve model performance, it has been pointed out by Longpre et al. (46) that some models may prioritize parametric knowledge over the input provided. The result is a model that prefers to work via parametric knowledge, generating an excess of information in the output. In our experience, the ACS sometimes refers to its parametric knowledge to provide a more specific classification, trying to predict details that are not always present in the report or which may be even incorrect.

As anticipated, reports evaluated by the ACS validation were obtained from generated synthetic data based on occupational medicine reports. On the one hand this solves legal and ethical implications in this study, but on the other hand this limits its real-world validity. Indeed, synthetic reports are, for example, free of any grammatical or typing errors. Such errors contained in the real texts can sometimes change the meaning of a word or even an entire sentence. Moreover, in real- world, each professional uses his or her own technical language, with various synonyms that also change over time, even in some cases introducing new terms before they are properly codified. As a result, validating the model only on grammatically “perfect” reports does not allow the evaluation of the ACS's ability to recognize semantic relationships under more complex conditions.

#### 4.1.2 Potential opportunities

In light of the experience described, there are several opportunities to improve the ACS, considering the potential use in RDs, with the final goal of implementing and optimizing this coding approach for disease registries.

A clear opportunity is to adopt and test a more updated classification system, such as ICD-11, which encompasses approximately 17,000 stem codes and 120,000 codable terms. This system is supported by a sophisticated coding algorithm, enabling it to interpret over 1.6 million terms (47). ICD-11 has a fully digital modular, flexible, and combinatorial approach designed to manage fine-grained details for EHRs, research and artificial intelligence (48). Additionally, an ACS could benefit from other coding sources, particularly in RDs scenario (49). Several ontologies and nomenclatures are used daily by rare disease experts to describe the clinical, treatment, and genetic aspects of this vast group of conditions. A multi-source approach that incorporates Orphanet Rare Disease Ontology (ORDO - capturing relationships between diseases, genes and other relevant features) (50), Human Phenotype Ontology (HPO - providing a standardized vocabulary of phenotypic abnormalities encountered in human disease) (51), and the Human Genome Variant Society (HGVS - nomenclature for describing DNA, RNA, and protein sequence variants) (52) and possibly a few more, would promote an even more accurate system for describing and detailing features of rare patients and monitoring disease evolution (53).

Implementing a more updated classification and/or a multi-ontology approach in an ACS will also improve interoperability among different institutions, research centers, and healthcare providers, promoting collaborations and shared research projects. This method of managing codes enables researchers and clinicians to advance both the semantic interoperability and the FAIR principles (54), which are key aspects in biomedicine and in RDs. Data federation in a trustworthy space may allow RDs experts to merge and integrate standardized datasets for secondary use of data, also taking advantage of AI tools in the process. This is particularly relevant in rare diseases, since data is scarce by nature, and frequently scattered and disorganized across separate databases.

From this perspective the use of properly coded information is also supported by a European initiative promoting the European Health Data Space (EHDS) (55, 56), a health-specific ecosystem comprising rules, common standards and practices, infrastructures and a governance framework. The EHDS aims to empower European citizens to have better control of their digital health data, to foster a unique market for technological systems and ensure a secure use of health data for research and regulatory purposes (56–58). In fact, to date the EHDS has been already considered a valuable space for primary use of orphan disease and rare cancer data (59). Nevertheless, the European commission recognizes the crucial role of EHDS in cross-border research and secondary use of data (55).

The introduction of a ACS has the potential to support the FAIRification process, by enabling the findability, accessibility, interoperability and reusability of data, through the use of shared, scientifically recognized coding classifications. In conjunction with an appropriate trustworthy space, such as EHDS, this may facilitate registry data integration and support broader data collection, in terms of sample size – a crucial aspect in RDs - and data types. Although the present study focused on single clinical reports, the approach of using an ACS could be expanded to cover more complex healthcare and research documents - including entire EHRs – as well as other data sources, such as demographic information, molecular evaluations, family history and imaging. Simplifying the input process is widely considered fundamental to tackle some long-standing challenges, for updating data, improving its quality and promoting sustainability in disease registries scenario (60, 61).

Of the various improvements that could be applied to an ACS, the introduction of a reliability scorer would, in our opinion, address some of the issues encountered in the performed study. This tool could define an evaluation metric to measure the quality of output across dimensions such as correctness and groundedness. As is frequently the case with Large Language Models (LLMs), coding system evaluation metrics can be implemented to measure the reliability and relevance of the answer, the semantic similarity and the model hallucinations, producing more quantitative, reliable and accurate results (62). This validation strategy would mitigate potential bias and encourage data users to pay additional attention to more prone-error coding, as mentioned by several studies in medical research (63, 64).

Another potentially beneficial approach that could mitigate code selection bias is the selection of superior hierarchical codes in specific cases. It is evident that a number of classifications, ontologies and standards exhibits a tree structure (41, 65). Consequently, in the absence of a specific detailed code, the ACS could select a superior code, moving from the leaf (child) to the twig (parent) term, instead of forcing a too detailed code. This approach is delicate in nature, as it carries the risk of diminishing the precision of the information, thereby introducing an element of uncertainty into the evaluation process of those who subsequently utilize the data. However, this would allow obtaining results that are more consistent with the input provided, limiting the prioritization of parametric knowledge in the processing of the output.

## 5 Conclusion

The ACS has demonstrated an acceptable level of accuracy, despite the limitations that the ICD-9-CM classification currently has in comparison with its successors. Further investigation is needed to in-depth evaluate some critical points of the ACS. In fact, analyses conducted on a larger dataset could shed light on discrepancies in the coding of diagnoses and procedures and on aspects such as overreliance on non-specific residual codes and the tendency to generate details not present. The application of an ACS in rare disease registries, despite the specific challenges involved, may represent a promising approach to simplify and streamline the time-consuming process of manual coding.

## Data Availability

The raw data supporting the conclusions of this article will be made available by the authors, without undue reservation.
